# A Neural-Network-Based Approach to White Blood Cell Classification

**DOI:** 10.1155/2014/796371

**Published:** 2014-01-30

**Authors:** Mu-Chun Su, Chun-Yen Cheng, Pa-Chun Wang

**Affiliations:** ^1^Department of Computer Science & Information Engineering, National Central University, Jhongli 32001, Taiwan; ^2^General Hospital, Taipei 10656, Taiwan

## Abstract

This paper presents a new white blood cell classification system for the recognition of five types of white blood cells. We propose a new segmentation algorithm for the segmentation of white blood cells from smear images. The core idea of the proposed segmentation algorithm is to find a discriminating region of white blood cells on the HSI color space. Pixels with color lying in the discriminating region described by an ellipsoidal region will be regarded as the nucleus and granule of cytoplasm of a white blood cell. Then, through a further morphological process, we can segment a white blood cell from a smear image. Three kinds of features (i.e., geometrical features, color features, and LDP-based texture features) are extracted from the segmented cell. These features are fed into three different kinds of neural networks to recognize the types of the white blood cells. To test the effectiveness of the proposed white blood cell classification system, a total of 450 white blood cells images were used. The highest overall correct recognition rate could reach 99.11% correct. Simulation results showed that the proposed white blood cell classification system was very competitive to some existing systems.

## 1. Introduction

The microscopic inspection of blood smears provides diagnostic information concerning patients' health status. The inspection results of the differential blood count reveal a wide range of important hematic pathologies. For example, the presence of infections, leukemia, and some particular kinds of cancers can be diagnosed based on the results of the classification and the count of white blood cells. The traditional method for the differential blood count is performed by experienced operators. They use a microscope and count the percentage of the occurrence of each type of cell counted within an area of interest in smears. Obviously, this manual counting process is very tedious and slow. In addition, the cell classification and counting accuracy may depend on the capabilities and experiences of the operators. Therefore, the necessity of an automated differential counting system becomes inevitable.

There are two kinds of techniques to implement an automated differential counting system. While the first and the dominant technique is based on the flow cytometry, the other technique is based on image processing. There are many commercially available systems which adopt the flow cytometry technique for counting cells. The flow-cytometry-based systems have an advantage (i.e., they can offer high throughput), but they suffer from one drawback (i.e., they cannot produce the images of the blood samples for further chance of verification in case some abnormal conditions were detected). Recently, several different approaches to implement an image-processing-based white blood cell recognition system have been proposed [1–11].

White blood cells can be categorized into several classes according to the morphology of their contours and their nuclei. The classification of white blood cells usually involves the following three stages: (1) the segmentation of a white blood cell from a smear image, (2) the extraction of effective features, and (3) the design of a classifier. For example, Young adopted four features and the minimum distance classifier for classifying 5 types of cells [4]. Sheikh et al. used wavelet transform coefficients and artificial neural networks for recognizing the white blood cell, the red blood cells, and platelets [8]. Bikhet et al. selected 10 features and adopted the minimum-distance classifier for constructing an automatic classification system which achieved the rate of 91% correct classification for a database consisting of 71 white blood cells [6]. Piuri and Scotti proposed an automatic detection and classification system based on 23 morphological features and a neural classifier [7]. A classification system based on eigen-cell and parametric features was proposed in [5]. A system which achieved a classification rate of 77% for classifying bone marrow white blood cells was reported in [9]. Nilufar et al. proposed a classification system based on joint histogram-based features and a support vector machine [10]. Osowski et al. presented the application of a genetic algorithm and a support vector machine to the recognition of bone marrow blood cells [11]. Rezatofighi et al. adopted morphological features and textural features extracted by local binary pattern (LBP) and then trained two types of neural networks for classification [1]. Tabrizi et al. adopted principal component analysis for features selection and used a learning vector quantization neural network for classifying 5 types of white blood cells [2]. Ghosh et al. fed four statistical significant features to Naïve Bayes classifier for classifying five types of white blood cells with 83.2% overall accuracy [3]. Each approach has its own considerations for adopting what kinds of features and classifier.

To a certain extent, the performance of an automatic white blood cell classification system depends on a good segmentation algorithm for segmenting white blood cells from their background. There are many different approaches (e.g., clustering [8], thresholding [5, 6, 10], morphological operator [11, 12], Gram-Schmidt orthogonalization method [1], edge detection [13], region growing [14], watershed [15], colors [16–18], and support vector machine (SVM) [19]) to segment white blood cells from the background. Each approach has its advantages and disadvantages. For example, the conventional color-based methods and the thresholding method are simple but are not able to accurately segment the white blood cells from the background. Some approaches (e.g., the SVM method and the region growing method) can provide reasonably accurate segmentation results, but they are either costly to be implemented or require high computational resources. A review on some of the general segmentation methods can be found in [20]. While some color-based segmentation methods (e.g., [17]) were directly conducted on the RGB color space, some approaches (e.g., [16, 18]) adopted the *HSI* color space (especially on the *S* component). In general, the *S*-component-based methods outperformed the RGB-based methods. In [16], the accuracy performance varied from 98.0% to 99.54% for the acute myeloid leukemia type and from 94.24% to 99.13 for the acute lymphocytic leukemia type.

In this paper, we propose a new approach to implementing an automatic white blood cell classification system. First of all, we try to identify the color characteristics of the pixels of the nucleus and granule of cytoplasm of white blood cells in the *HSI* color space. Based on the found discriminating region and a morphological process, we can segment a white blood cell from a smear image. In the following, we extract three kinds of features (i.e., geometrical features, color features, and LDP-based texture features) from the segmented cell region. These features are fed into three different neural networks for classifying five types of the white blood cells. The proposed system will be introduced in Section 2. The experimental results are given in Section 3. Finally, Section 4 concludes the paper.

## 2. The Proposed Automatic White Blood Cell Classification System

The proposed automatic white blood cell classification system involves the following three stages: (1) the segmentation of a white blood cell, (2) the extraction of effective features, and (3) the design of a classifier.

### 2.1. Stage 1. The Segmentation

**Figure 1:**
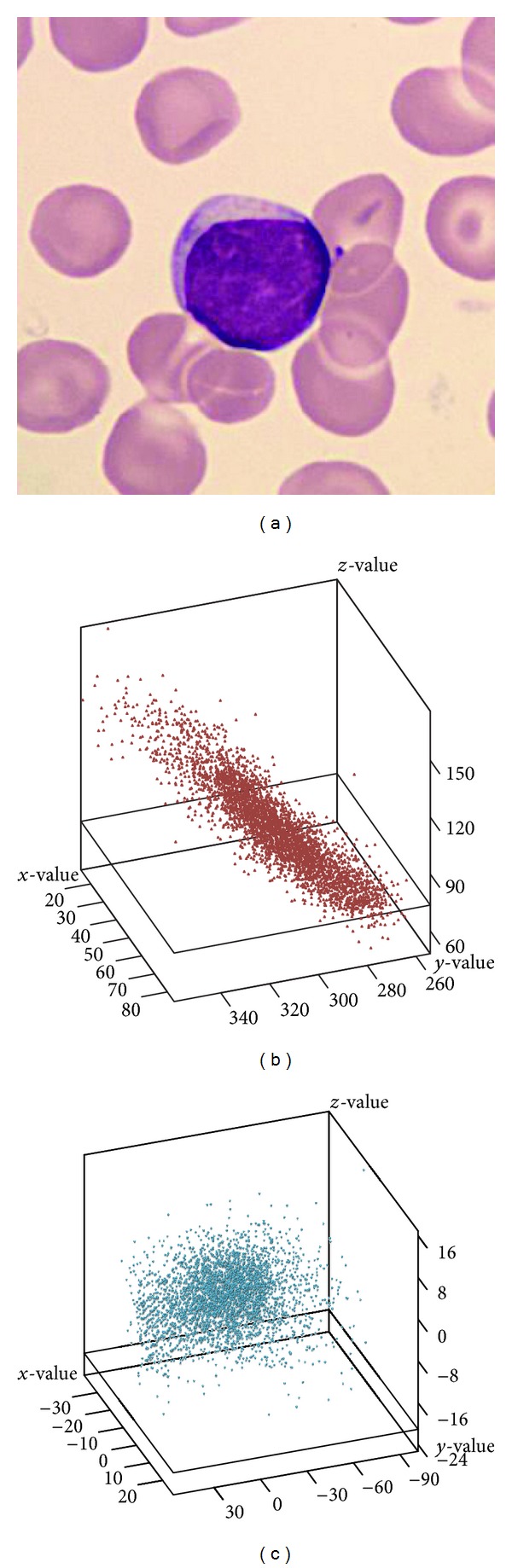
Blood cell images. (a) The original image. (b) The scatter plot of the collected pixels of the white blood cells in the *HSI* color space. (c) The scatter plot rotated to a new coordinate system.

In this paper, we propose a new color-based approach to segment five types of white blood cells from their background. This new color-based method is based on the idea of constructing a discriminating region for the scatter plot of pixels belonging to white blood cells. Via checking whether a pixel lies inside the discriminating region, white blood cells can be effectively segmented from the background. The database of the white blood cells used in this paper was downloaded from the CellaVision Competency Software Databases which contain slides stained with either a May Grünwald Giemsa (MGG) or a Wright staining protocol [21].  Figure 1(a) shows a sample of the images. First of all, an expert was asked to manually segment the white blood cells from the background to provide ground truth information. We then collected a set of pixels belonging to white blood cells.  Figure 1(b) shows the scatter plot of these collected pixels on the *HSI* color space. Obviously, most of these pixels are clustered inside a rotated ellipsoid. We can use the principal component analysis (PCA) method to find the principal axes of these pixels on the original *HSI* space and then use the principal axes information to rotate the ellipsoid to be parallel to the new coordinate system as shown in Figure 1(c). Finally, the discriminating region for white blood cell pixels can be described by the following equations:

(1) $$\begin{array}{c} \left[\begin{array}{c} H^{\prime }\\ S^{\prime }\\ I^{\prime } \end{array}\right]=R\left[\begin{array}{c} H\\ S\\ I \end{array}\right]=\left[\begin{array}{ccc} R_{11} & R_{12} & R_{13}\\ R_{21} & R_{22} & R_{23}\\ R_{31} & R_{32} & R_{33} \end{array}\right]\left[\begin{array}{c} H\\ S\\ I \end{array}\right]\\ \frac{{\left(H^{\prime }-C_{H}\right)}^{2}}{r_{H}^{2}}+\frac{{\left(S^{\prime }-C_{S}\right)}^{2}}{r_{S}^{2}}+\frac{{\left(I^{\prime }-C_{I}\right)}^{2}}{r_{I}^{2}}\leq 1, \end{array}$$

where the matrix *R* is a 3 × 3 rotation matrix, the vector (*H*′, *S*′, *I*′) is the rotated version of the original color vector (*H*, *S*, *I*), the parameter (*C*_*H*_, *C*_*S*_, *C*_*I*_) is the rotated ellipsoid center, and the parameters *r*_*H*_, *r*_*S*_, and *r*_*I*_ are the three semiprincipal axes of length. The appropriate values of the rotation matrix, the three semiprincipal axes, and the center may vary with the stain used to generate the blood cell images.

**Figure 2:**
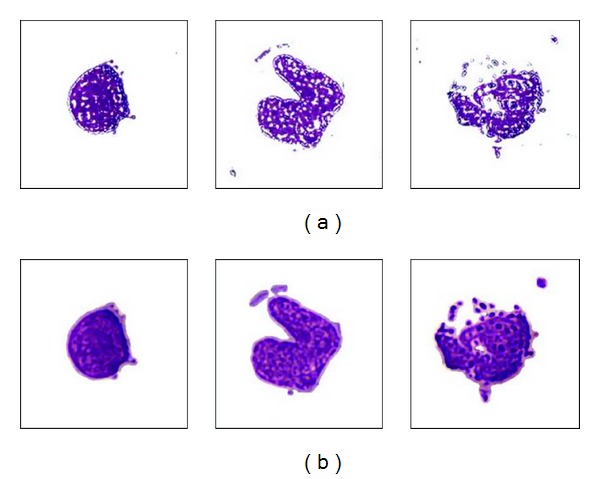
The detected white blood cell. (a) The detected cell based on the result of (1). (b) The detected cell after the morphological operators.

We may then use the ellipsoidal equations defined in (1) to verify whether a pixel belongs to a white blood cell. If the *HSI* information of a pixel satisfies (1), then it will be claimed to be a white blood cell pixel. Some detected cells images are shown in Figure 2(a). Obviously, the segmented white blood cells are broken and noisy; therefore, we still need to adopt some morphological operators (e.g., dilation, closing, and a 7 × 7 median filter) to remove unwanted small noisy regions and fill holes in the detected cell region as shown in Figure 2(b).

### 2.2. Stage 2. Feature Extraction

The feature extraction plays an important role in the performance of an automatic white blood cell classification system. Most of the existing methods adopt the following features such as geometrical features (e.g., area, radius, perimeter, convex area, major axis length, compactness, and orientation), textural features (e.g., momentum, contrast, entropy, and kewness), and color features (e.g., color distribution and histogram). For example, Piuri and Scotti greatly depended on the geometrical features [7]. Tabrizi et al. adopted both geometrical features and textural features [2]. Osowski et al. integrated all those three kinds of features to form 164 features for their classifiers [11].

In this paper, three kinds of features are extracted for classification. The first kind of features is the geometrical features consisting of the area feature, Area, the length feature, Length_var_, and the compactness feature, Comp. The area feature, Area, is the amount of pixels which belong to the segmented cell region. As for the last two features, Length_var_ and the compactness, Comp, are computed as follows:

(1)  Length_var_:

(2) $$\begin{array}{c} {\text{Length}}_{\text{var}}=\frac{1}{N_{b}}\overset{N_{b}}{\underset{i=1}{\sum }}{\left({\text{length}}_{i}-\bar{\text{length}}\right)}^{2}, \end{array}$$

where length_*i*_ represents the length between the *i*th pixel on the cell boundary and the cell center. The parameter $\overset{-}{\text{length}}$ represents the mean of those lengths. And

(2)  Comp:

(3) $$\begin{array}{c} \text{Comp}=\frac{\text{the perimeter of the cel}{\text{l}}^{\text{2}}}{\text{the area of the cell}}. \end{array}$$

The color features, Hue_var_, Saturation_var_, and Intensity_var_, are the three variances of the hue, saturation, and intensity components of the pixels belonging to the white cell.

As for the textural features, there are different ways to compute textural features. For example, Osowski et al. computed the angular second momentum, contrast, entropy, and so forth [11]. Rezatofighi et al. introduced the textural features extracted by local binary pattern (LBP) and cooccurrence matrix [1]. They found that the performance of the cooccurrence matrix-based features had better performance in comparison with the LBP-based features; however, the computational time for computing the cooccurrence matrix-based features was significantly higher than the time required by the computations of the LBP-based features.

Based on the aforementioned discussions, we proposed the use of the local-directional-pattern- (LDP-) based features to make a tradeoff between the computational time and the performance. The LBP operator, a gray-scale invariant texture primitive, was originally proposed by Ojala et al. [22]. Later on, the local-directional-pattern- (LDP) was proposed to overcome the disadvantages of LBP (e.g., nonmonotonic illumination variation and random noise). The LDP is an eight bit binary code and is obtained by computing the relative edge response value of a pixel in all eight directions at each pixel position [23].

**Figure 3:**
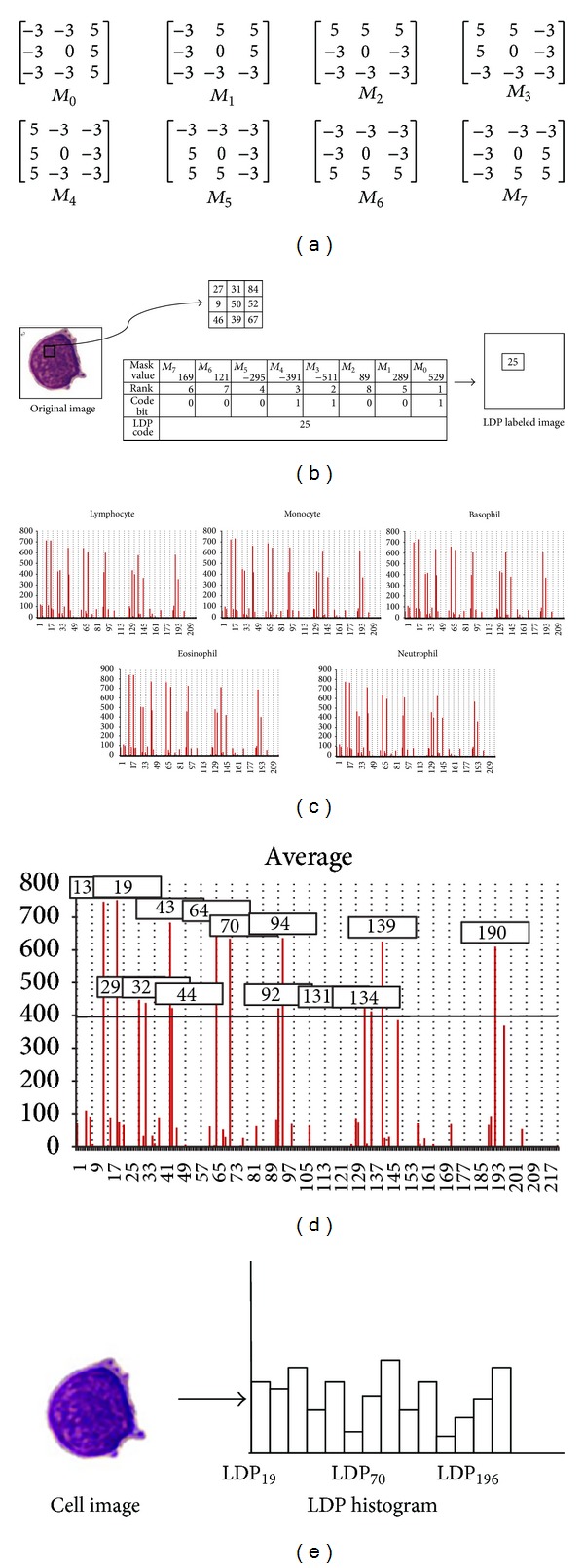
LDP-based features. (a) The Kirsch edge masks used for detecting the 8 directions. (b) The LDP code. (c) The LDP histograms with 218 bins. (d) The average LDP histogram. (e) The reduced LDP histogram with 14 bins for representing the cell image.

In our system, we were interested at the three most prominent directions in order to generate the LDP. Figures 3(a)-3(b) illustrate an example of transforming a 3 × 3 windowed image into a LDP code. We then use the LDP operator to transform a gray image to a LDP labeled image where the value of each pixel is the computed LDP code corresponding to the pixel at the same position at the original gray image as shown in Figure 3(b). In the following, we can use a so-called “LDP histogram” with 218 bins (i.e., from 00000111(= 7) to 11100000(=224)) to represent this LDP labeled image as shown in Figure 3(c). The LDP histogram represents the relative frequency of occurrence of the various LDP codes in the LDP labeled image.  Figure 3(d) shows the average LDP histogram computed from 60 cell images consisting of 5 types of white blood cell images. We found that bins, 19, 25, 35, 38, 49, 50, 70, 76, 98, 100, 137, 140, 145, and 196, are the 14 bins with the 14 largest values which are larger than a threshold, 400. These 14 bins accounted for a very large proportion of the relative frequency of occurrences. Therefore, we decided to use a reduced histogram with those 14 especially important bins to represent a LDP labeled image as shown in Figure 3(e).

In total, we have extracted 20 features for the classification purpose, Area, Length_var_, Comp, Hue_var_, Saturation_var_, Intensity_var_, LDP_19_, LDP_25_, LDP_35_, LDP_38_, LDP_49_, LDP_50_, LDP_70_, LDP_76_, LDP_98_, LDP_100_, LDP_137_, LDP_140_, LDP_145_, and LDP_196_.

### 2.3. Stage 3. Classification

After the set of features have been extracted, we proceed to the design of classifiers. Three different kinds of neural-network-based classifiers were employed in the design of classifiers.

#### 2.3.1. The Multilayer Perceptron

Multilayer perceptrons (MLPs), one of the most popular neural networks, have been applied successfully to solve many difficult and diverse problems by using the well-known backpropagation algorithm to train them [24]. The performance of a trained MLP depends on its architecture, initial weights, and the number of training epochs. From many simulations, an MLP with the architecture 20 × 12 × 10 × 5 was constructed.

#### 2.3.2. The Support Vector Machine

The support vector machine (SVM) is another widely adopted neural network [24]. It is a linear machine with powerful learning ability and good generalization capability. The design of a successful SVM classifier involves the choice of the so-called kernel functions. In our system, we employed the use of the radial-basis functions (RBFs). The number of RBFs and their centers are automatically set by the number of support vectors and their values.

#### 2.3.3. Hyperrectangular Composite Neural Networks

The class of hyperrectangular composite neural networks (HRCNNs), a kind of hybrid networks developed by our previous work [25–28], integrates the paradigms of neural networks with the rule-based approach. The values of the synaptic weights of a trained HRCNN can be interpreted as a set of crisp If-Then rules. In addition, a specially designed training algorithm can achieve 100% correct recognition rate for the training set [25–28]. The mathematical description of a two-layer HRCNN with *J* hidden nodes is given as follows:

(4) Out(x_)=f(∑j=1JOutj(x_)−η),                      Outj(x_)=f(netj(x_)),netj(x_)=∑i=1nf((Mji−xi)(xi−mji))−n,f(y)={1if y≥00if y<0,

where *M*_*ji*_ and *m*_*ji*_ ∈ *ℜ* are adjustable synaptic weights of the *j*th hidden node, $\underline{x}={\left(x_{1},\ldots ,x_{n}\right)}^{T}$is an input pattern, *n* is the dimensionality of input variables, *η* is a small positive real number, and $\text{Out}(\underline{x}):{ℜ}^{n}\to \{0,\;1\}$ is the output function of a two-layer HRCNN with *J* hidden nodes.

## 3. Experimental Results

**Figure 4:**
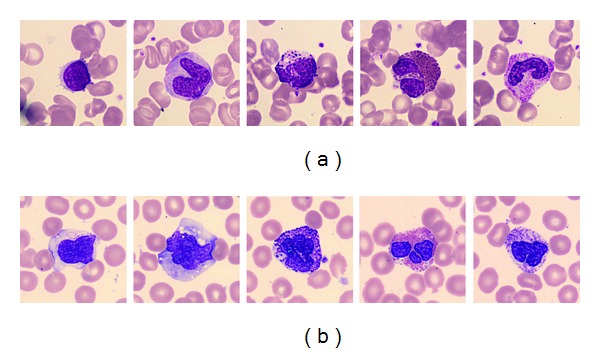
Samples of white cell images from the CellaVision Competency Software Databases. From left to right, lymphocyte, monocyte, eosinophil, basophil, and neutrophil. (a) Data set 1. (b) Data set 2.

The database of the white blood cells used in the experiments was downloaded from the CellaVision Competency Software Databases which contain slides stained with either a May Grünwald Giemsa or a Wright staining protocol [21]. The databases contain 9-10 slides each having approximately 100 white blood cell images and a large RBC overview image for RBC characterization. There are five types of white blood cells (e.g., lymphocyte, monocyte, eosinophil, basophil, and neutrophil). There were 450 white blood cell images used in our experiments. These 450 images came from two different data sets. The difference between these two data sets was the colorant used to stain cells.  Figure 4 shows some examples from the two data sets. The color of the white blood cells which came from data set 2 looks more near purple than the cells from data set 1.  Table 1 tabulates the number of cell images for each type of white cells.

### 3.1. Experiment One: The Cell Segmentation

In this experiment, we would like to test the performance of the proposed segmentation algorithm introduced in the first stage of the proposed automatic white blood cell classification system. Since the preparation of staining procedure and blood concentration of individual may result in the inconsistency of color in cell images, we constructed one discriminate region of white blood cell tones particularly suitable for each data set. The two discriminating regions were constructed based on 500 and 800 pixels which were randomly selected from the images from the two data sets, respectively. The found discriminating region for each data set was given as follows:

for data set 1,

(5) $$\begin{array}{c} \left[\begin{array}{c} H^{\prime }\\ S^{\prime }\\ I^{\prime } \end{array}\right]=\left[\begin{array}{ccc} -0.6590 & 0.3518 & -0.7135\\ 0.7527 & 0.5438 & 0.3711\\ -0.2574 & 0.7620 & 0.5943 \end{array}\right]\left[\begin{array}{c} H\\ S\\ I \end{array}\right]\\ \frac{{\left(H^{\prime }-6.042\right)}^{2}}{52.645_{}^{2}}+\frac{{\left(S^{\prime }-2.916\right)}^{2}}{17.274_{}^{2}}+\frac{{\left(I^{\prime }-0.252\right)}^{2}}{11.154_{}^{2}}\leq 1; \end{array}$$

for data set 2,

(6) $$\begin{array}{c} \left[\begin{array}{c} H^{\prime }\\ S^{\prime }\\ I^{\prime } \end{array}\right]=\left[\begin{array}{ccc} -0.3683 & 0.5550 & -0.7459\\ 0.8238 & 0.5667 & 0.0149\\ -0.4309 & 0.6090 & 0.6659 \end{array}\right]\left[\begin{array}{c} H\\ S\\ I \end{array}\right]\\ \frac{{\left(H^{\prime }+16.433\right)}^{2}}{56.761_{}^{2}}+\frac{{\left(S^{\prime }+0.0512\right)}^{2}}{40.027_{}^{2}}+\frac{{\left(I^{\prime }+8.735\right)}^{2}}{21.894_{}^{2}}\leq 1. \end{array}$$

The evaluation of the proposed segmentation algorithm was based on the computations of the sensitivity and specificity rates:

(7) sensitivity=number of true positives×(number of true positives  +  number of false negatives)−1,specificity=number of true negatives×(number of true negatives  +  number of false positives)−1.

While the sensitivity rate relates to the test's ability to identify positive results (i.e., white blood cell pixels), the specificity rate relates to the test's ability to identify negative results (i.e., background pixels).  Table 2 tabulates the segmentation results. For the four types, lymphocyte, monocyte, eosinophil, and neutrophil, the sensitivity and specificity rates were all above 0.97. It indicated that the segmentation results were good. As for the basophil type, the specificity rate was still high (i.e., 0.993), but the sensitivity rate was only 0.794 (for data set 1) and 0.867 (for data set 2). It indicated that the basophil type was a little undersegmented. We found that the low sensitivity may be due to the large amount of red granules in a basophil. The segmentation performance was not 100% correct; however, the classification performance could be high if the most important regions of the cells were correctly segmented. The classification performance was validated at the second experiment.

### 3.2. Experiment Two: The Classification Comparisons

The 450 images were split into a training set consisting of 299 images and a testing set consisting of 151 images. The classification results for these three types of classifiers were tabulated in Table 3 based on the average of the classification rates of all classes. While the SVM and the HRCNN could achieve 100% correct rate for the training set, the MLP could reach the highest rate for the testing data set. The MLP outperformed the other two classifiers based on the comparisons of the overall correct rate.

The comparisons of our proposed white cell recognition system with other white cell classification systems were shown in Table 4. Those existing systems constructed their own databases instead of using a public database as we did. The rightest column shows the number of images used in their experiments. Among these systems, we used the largest amount of images to evaluate the proposed system. In addition, based on the comparisons of the overall rate, our proposed system incorporated with a trained MLP achieved the highest correct rate.

Most of the systems shown in Table 4 did not mention the information about the computational time. While Rezatofighi et al. [1] reported that it took 16 minutes for the differential counting of 100 white cells with the image size 720 × 576 on a Pentium-4 PC at 3.2 GHz with 1 GB RAM, our system took 0.88 seconds (from segmentation to classification) to classify a cell image with the size 360 × 360 on a Pentium-4 PC at 2.6 GHz with 2 GB RAM.

## 4. Conclusions

In this paper, we proposed a new segmentation algorithm for segmenting a white cell from a smear image. This segmentation algorithm is based on finding a discriminating region of white blood cell tones in the *HSI* color space. The found discriminating region can be described by a 3D ellipsoid. Then we proposed the use of 20 features consisting of 3 geometrical features, 3 color features, and 14 LDP features. Finally, three different neural-network-based classifiers were adopted for classifying white blood cells into one of the five types. Compared to other systems tabulated in Table 4, our proposed system incorporated with a trained MLP could reach the highest performance. The performance was evaluated on a public database.

## Figures and Tables

**Table 1:** The number of cell images for each kind of white cells.

	Lymphocyte	Monocyte	Basophil	Eosinophil	Neutrophil	Total
Set 1	12	12	12	12	12	60
Set 2	83	31	2	5	269	390

Total	95	43	14	17	281	450

**Table 2:** The segmentation results.

Data set	1		2	
Cell type	Sensitivity	Specificity	Sensitivity	Specificity
Lymphocyte	0.995	0.994	1.000	0.986
Monocyte	0.997	0.988	0.999	0.983
Basophil	0.970	0.992	0.999	0.970
Eosinophil	0.794	0.994	0.867	0.993
Neutrophil	0.990	0.990	1.000	0.978

Average	0.949	0.992	0.973	0.982

**Table 3:** The classification results of the three neural-network-based classifiers.

Classifier	Training set	Testing set	Overall
MLP	99.67%	98.01%	99.11%
SVM	100.0%	92.72%	97.55%
HRCNN	100.0%	66.90%	88.89%

**Table 4:** The comparisons of the classification rates among different classification systems.

Method	Number of types	Segmentation	Classifier	Overall rate	Number of images
Ours	5	Discriminating region	MLP	99.11%	450
Ours	5	Discriminating region	SVM	97.55%	450
Ours	5	Discriminating region	HRCNN	88.89%	450
Rezatofighi et al. [1]	5	Gram-Schmidt orthogonalization and snake	SVM	86.10%	400
Tabrizi et al. [2]	5	Gram-Schmidt orthogonalization and snake	LVQ	94.10%	400
Ghosh et al. [3]	5	Watershed	Bayes classifier	83.2%	150
Young [4]	5	Histogram threshold	Distance classifier	92.46%	199
Yampri et al. [5]	5	Automatic thresholding and adaptive contour	Minimized error	96.0%	100
Bikhet et al. [6]	5	Entropy threshold and iterative threshold	Distance classifier	90.14%	71
Piuri and Scotti [7]	5	Opening and Canny edge detector	KNN, FF-NN, and RBF	92%~82%	113
